# Unraveling the K^+^ transporter gene family in rice: evolution, subcellular localization, K^+^ uptake activity, and transcriptional responses to salt stress

**DOI:** 10.3389/fpls.2026.1980352

**Published:** 2026-10-15

**Authors:** Tongyuan Yu, Jiadong Ren, Yue Yuan, Yuan Xue, Jie Liang, Zheyu Xu, Kejia Zhu, Mengying Jiang, Yunxia Fang, Xiaoqin Zhang, Yijun Meng, Zhonghua Chen, Quanxiang Tian, Dawei Xue

**Affiliations:** 1 College of Life and Environmental Sciences, Hangzhou Normal University, Hangzhou, China; 2 School of Agriculture, Food and Wine, Waite Research Institute, University of Adelaide, SA, Glen Osmond, Australia

**Keywords:** bioinformatics, HAK/KUP/KT, K+ uptake activity, rice, salt stress

## Abstract

Rice is a global staple food crop, and its yield is seriously limited by soil salinity. The high-affinity K^+^ transporter/K^+^ uptake permease/K^+^ transporter (HAK/KUP/KT) family of potassium transporters plays a pivotal role in K^+^ homeostasis and plant salt tolerance. However, their systematic characterization and functional relevance to salt tolerance in rice have not been clarified. Here, we conducted genome-wide identification and structural characterization of the HAK/KUP/KT family, assessed their expression profiles under salt stress, and examined their K^+^ transport function. In total, 27 *OsHAK* genes were identified in the rice genome, most of which exhibited salt-responsive expression. Yeast functional complementation assays confirmed that OsHAK11, OsHAK13, OsHAK23, OsHAK24, OsHAK25, and OsHAK26 can mediate K^+^ absorption. Among them, OsHAK11 and OsHAK25 showed relatively strong K^+^ uptake ability. Subcellular localization analysis revealed that OsHAK11 and OsHAK25 were located in the plasma membrane. Our research systematically analyzed the sequence characteristics of the rice HAK gene family, examined their transcriptional responses to salt stress, and further evaluated the K^+^ uptake capacity of HAK proteins. These results provide a foundation for further elucidating the function of OsHAKs in plant salt tolerance.

## Introduction

Salt stress represents a major environmental limiting factor that severely restricts crop growth and productivity. The detrimental effects of salt stress on plants primarily occur due to osmotic stress and sodium ion (Na^+^) toxicity. Excessive Na^+^ influx into plant cells competes with potassium ion (K^+^) uptake and can induce K^+^ efflux, thereby disrupting the intracellular ion balance. Consequently, maintaining K^+^ homeostasis under saline conditions is critical for plant survival. As an essential macronutrient, potassium serves as a cofactor in numerous enzymes, plays a vital role in osmoregulation, and functions as a central component in various metabolic processes. K^+^ uptake and translocation are mainly mediated by specific transporters and channels (Wang and Wu, 2013).

The High Affinity K^+^ Transporters/K^+^ Uptake Permease/K^+^ Transporters (*HAK*/*KUP*/*KT*) gene is essential for maintaining intracellular K^+^ homeostasis under salt stress (Wang et al., 2013; Wang and Wu, 2017; Feng et al., 2020; Wang et al., 2022; Morita et al., 2023; Shen et al., 2023). In *Arabidopsis*, *AtHAK5* plays a critical role in K^+^ uptake and plant growth under salt stress. However, its transcription is inhibited by salt stress (Nieves-Cordones et al., 2010). Further studies have revealed that the *AtHAK5* promoter is directly bound by multiple transcription factors, including ARF2, DDF2, JLO, TFII, and bHLH121, which exert distinct regulatory effects: ARF2 represses AtHAK5 transcription, whereas DDF2, JLO, TFII, and bHLH121 activate its expression (Kim et al., 2012; Hong et al., 2013; Zhao et al., 2016). In previous research, AtKUP2, localized to the plasma membrane and mitochondria, reduced the Na^+^/K^+^ ratio and enhanced the salt tolerance of plants when overexpressed in rice (Rajappa et al., 2020). The *AtKUP12* mutation increased K^+^ absorption and salt tolerance (Zhang et al., 2021). In rice, *OsHAK1*, *OsHAK5*, and *OsHAK21* are critical for K^+^ acquisition, and their loss increases the plant Na^+^/K^+^ ratio and salt sensitivity (Yang et al., 2014; Chen et al., 2015; Shen et al., 2015; He et al., 2019). At the transcriptional level, *OsHAK1* expression is repressed by the AP2/ERF transcription factor KTR1 but activated by KTR2, whereas OsHAK5 transcription is negatively regulated by the brassinosteroid-responsive transcription factor OsBIM1 (Li et al., 2025; Chen et al., 2026). OsHAK21 interacts with OsCYB5-2, which enhances the apparent affinity of OsHAK21 for K^+^ binding and increases its transport activity (Song et al., 2021). *OsHAK16* overexpression improves salt tolerance by reducing the plant Na^+^/K^+^ ratio, while *OsHAK9* limits K^+^ efflux and promotes germination via gibberellin regulation (Zeng et al., 2024). Conversely, OsHAK2 and OsHAK12 mediate sodium transport. The overexpression of *OsHAK2* increases Na^+^ accumulation and sensitivity, and its transcription is regulated by OsBBX17 (Morita et al., 2023; Shen et al., 2024). However, the *OsHAK12* mutation leads to shoot Na^+^ overaccumulation and susceptibility (Zhang et al., 2021). Additional genes have also been reported to be involved in salt tolerance regulation, such as *ZmHAK4*, *SlHAK20*, *IbHAK1*, *MeHAK5*, *CeqHAK6*, and *CeqHAK11* (Zhang et al., 2019; Wang et al., 2022; Zhu et al., 2023; Luo et al., 2024; Zhang et al., 2025).

The *HAK* family is integral to potassium (K^+^) nutrition across plant species. In *Arabidopsis thaliana*, AtHAK5, AtKUP7, and AtKUP9 mediate K^+^ absorption. Their mutants exhibit low-potassium phenotypes such as yellowing of leaves (Gierth et al., 2005; Han et al., 2016; Zhao et al., 2016; Maierhofer et al., 2024; Šustr et al., 2024). Similarly, in rice, *OsHAK1* and *OsHAK16* are upregulated under K^+^ deficiency and are essential for efficient potassium absorption, while *OsHAK18* regulates K^+^ phloem loading and influences Na^+^ and sugar transport (Peng et al., 2023; Shen et al., 2023). The heterologous expression of *TaHAK13* complements the low-K^+^ sensitivity of *athak5* mutants, and the overexpression of TaHAK18 enhances root architecture and biomass in *Arabidopsis* under K^+^ limitation (Liu et al., 2025). Additional members such as SlHAK5 mediate high-affinity K^+^ uptake, and IbHAK11 maintains root growth by regulating K^+^ homeostasis (Zhang et al., 2020b; Templalexis et al., 2022; Zhu et al., 2023). In addition to participating in salt stress response and low potassium stress, *HAK* members, including *OsHAK1*, *OsHAK5*, and *OsHAK26*, are also involved in pollen development and yield regulation (Li et al., 2022).

Rice is one of the most important food crops in the world, but its yield has been severely limited by salt stress. Although it is known that the *HAK* gene plays a crucial role in K^+^ absorption and salt stress responses in various plant species, systematic analysis of its family-wide characterization, subcellular localization, transcriptional responses to salt stress, and potassium transport functions remain lacking in rice. In this study, we systematically identified *HAK* gene family members in rice and performed comprehensive analysis of their physicochemical properties, subcellular localization, nuclear localization signals, phylogenetic evolution, and promoter *cis*-acting elements. Furthermore, we investigated the transcriptional response of *OsHAK* genes to salt stress, as well as the K^+^ absorption capacity of *HAK* genes. These findings enhance our understanding of the *OsHAK* gene family and offer important perspectives for further functional studies aimed at improving salt tolerance through genetic approaches.

## Materials and methods

### Chromosome mapping, gene structure, and conserved sequence analysis

The genome information of *OsHAK* genes was obtained from the InterPro database (https://www.ebi.ac.uk/interpro/, accessed on June 14, 2024), based on the reported Arabidopsis *HAK* family members. Then, we acquired gene annotations from RiceData (https://www.ricedata.cn/gene/, accessed on June 15, 2024) and mapped chromosomal positions using TBtools-II v2.031 (Chen et al., 2023a). The coding sequences (CDSs) and corresponding genomic DNA of *OsHAK* genes were retrieved in FASTA format from Ensembl Plants (https://plants.ensembl.org/index.html, accessed on June 16, 2024). Gene structure features were visualized in TBtools-II v2.031. MEME Suite (http://meme-suite.org/, accessed on June 16, 2024) was employed for conserved motif analysis, with 12 motifs specified under default parameters. The resulting motifs were then annotated using TBtools-II v2.031.

### Protein sequence alignment, phylogenetic tree construction, gene duplication analysis, and homology analysis

We identified the protein sequences and PF numbers (PF02705) of the OsHAK family by searching for the reported HAK family gene symbols, and retrieved all protein sequences from Arabidopsis, Brachypodium, barley, and sorghum from the UniProt website (https://www.uniprot.org/). The conserved domains of OsHAK proteins were aligned using MEGA 11, which was also employed to construct phylogenetic relationships among *Oryza sativa* L. (*Os*), *Arabidopsis thaliana* (L.) Heynh. (*At*), *Brachypodium distachyon* (L.) P. Beauv. (*BRADI*), *Hordeum vulgare* L. (*Hv*), and *Sorghum bicolor* L. (*SORBI*). Fragment duplication events within this gene family were analyzed with TBtools-II v2.031 and visualized as Circos plots. Homology analysis of OsHAK proteins was performed using TBtools-II v2.031.

### Prediction of physicochemical properties, subcellular localization, secondary structure, and three-dimensional structure of proteins

Protein physicochemical properties were predicted using ExPASy ProtParam (https://web.expasy.org/protparam/, accessed on July 1, 2024), with sequences submitted in FASTA format and default parameters applied to obtain amino acid composition, theoretical pI, molecular weight, and hydrophobicity index. Subcellular localization was assessed via WoLF PSORT (https://wolfpsort.hgc.jp/, accessed on July 1, 2024) by selecting the “plant” option and inputting FASTA sequences, with the organelle receiving the highest score reported as the predicted location. Nuclear localization signals were predicted using NucPred (https://nucpred.bioinfo.se/nucpred/, accessed on July 1, 2024), where sequences were submitted through the “single protein” service and scores ≥0.8 were considered indicative of nuclear localization. Secondary structures were generated with SOPMA (https://npsa-prabi.ibcp.fr/, accessed on July 1, 2024) under settings (output width: 70, conformational states: 4, similarity threshold: 8, window width: 17). Three-dimensional protein models were constructed via SWISS-MODEL server (https://swissmodel.expasy.org/, accessed on July 1, 2024), using structural templates retrieved from the AlphaFold database. The modeling was performed by submitting target sequences in FASTA format, selecting the model with the highest GMQE score based on templates predicted by AlphaFold v2, and the confidence of the predicted structure was evaluated based on the per-residue pLDDT score provided by AlphaFold.

### Analysis of cis-acting elements in the promoter region of OsHAKs and protein–protein interaction network analysis

Promoter sequences (-2 kb upstream of start codons) for rice *OsHAK* genes were extracted from Ensembl Plants (http://plants.ensembl.org, accessed on August 3, 2024). *Cis*-acting elements within these promoters were predicted using PlantCARE (http://bioinformatics.psb.ugent.be/webtools/plantcare/html/, accessed on August 3, 2024) and visualized using TBtools-II v2.031. PPI networks of OsHAK proteins were constructed using the STRING database (https://cn.string-db.org/, accessed on August 3, 2024) and visualized in Cytoscape v3.7.2 to identify interacting partners.

### Analysis of OsHAK gene family expression profiles and transmembrane domains

Fertility-related expression profiles of the *Oryza sativa* L. spp. *japonica* were retrieved from RGAP (https://rice.uga.edu/expression.shtml, accessed on December 4, 2025). The expression levels of *OsHAK* genes across 11 organs and developmental stages were extracted, followed by cluster analysis using TBtools-II v2.031. Transmembrane domains were predicted using TMHMM 2.0 (https://services.healthtech.dtu.dk/service.php?DeepTMHMM, accessed on August 5, 2024) with “extensive, with graphics” output selected for protein sequence submissions.

### Yeast complementation experiment

Full-length CDSs of *AtKAT1*, *OsHAK11*, *OsHAK13*, *OsHAK23*, *OsHAK24*, *OsHAK25*, and *OsHAK26* were cloned into pYES2 yeast expression vector. Constructs were transformed into the potassium-uptake-deficient yeast strain CY162 (*MATa*, *ura3-52*, *trkl*Δ, *his3*Δ*200, his4-15, trk2*Δ*l::pCK64*) (Anderson et al., 1992) and plated on SD/-Ura medium with 50 mM KCl (PM2271, Beijing Coolaber Technology, Beijing, China). After 48 h of incubation at 30 °C, polymerase chain reaction (PCR)-verified colonies were inoculated into SD/-Ura liquid medium and cultured for 16 h at 30 °C. Cells were harvested at room temperature, washed extensively with sterile water to remove residual K^+^, and adjusted to OD_600_ = 1.0. Serial 10-fold dilutions were spotted (5 μl) onto AP medium (PM4030L, Beijing Coolaber Technology, Beijing, China) containing graded K^+^ concentrations. Growth phenotypes were documented following 72 h of incubation at 30 °C.

### Subcellular localization analysis

*OsHAK11* and *OsHAK25* CDSs (stop codon excluded) were cloned into pCAMBIA1307-GFP fusion vector. Recombinant plasmids were transformed into *Agrobacterium tumefaciens* GV3101. Bacterial cultures were activated twice to OD_600_ = 1.0, resuspended in infiltration buffer, and pressure-infiltrated into *N. benthamiana* leaves. Infiltrated leaves were returned to normal growth conditions(25 ± 2 °C,16 h light/8 h dark) for 48 h. The fluorescent signals were subsequently visualized using a Zeiss LSM 900 confocal microscope with an argon laser (488 nm for GFP excitation, 568 nm for mCherry excitation). Images were analyzed and processed using ZEN 3.9 (Zeiss).

### Plant growth and stress treatment

The rice material used in the experiments was the *japonica* cultivar Zhonghua 11 (ZH11), purchased from Weimi Biotechnology Co., Ltd. The rice seedlings was grown hydroponically in International Rice Research Institute nutrient solution (adjusted to pH 5.5–5.8 using KOH), which was renewed triweekly. Seedlings were maintained in growth chambers at 28 °C/70% relative humidity (RH) under a 14 h photoperiod and 24 °C/70% RH (10 h dark). To induce salt stress, two-leaf-stage seedlings were exposed to 140 mM NaCl-supplemented nutrient solution. After treatment for 5 h, tissues were harvested for gene expression analysis.

### RNA extraction and quantitative real-time PCR (qRT-PCR) analysis

The qRT-PCR primers used in this study were designed in Primer Premier 5, using *OsUBQ5* as the reference gene (**Supplementary Table 1**). Total RNA was extracted using the RNAprep Pure Plant Kit (Catalog No.: DP432, Tiangen Biotech Co., Ltd.) and reverse-transcribed using HiScript III RT SuperMix (+gDNA wiper) (Catalog No.: R323-01, Nanjing Vazyme Biotech Co., Ltd., Nanjing, China). Amplification was performed on the CFX96 Real-Time System (Bio-Rad, USA) under the following cycling conditions: 95 °C for 5 min; 38 cycles of 94 °C for 35 s, 63 °C for 40 s, and 72 °C for 20 s. Quadruplicate reactions were analyzed using the $2^{-\Delta \Delta Ct}$ method.

## Results

### Identification of the OsHAK gene family and analysis of its physicochemical properties, subcellular localization, and nuclear localization sequences

We identified 27 *HAK* genes distributed across eight chromosomes in the rice genome, with none found on chromosomes 5, 10, 11, and 12 (**Figure 1**). The physicochemical properties, subcellular localization, and nuclear localization signals of OsHAK proteins are presented in **Table 1**. The amino acid length of OsHAK proteins ranged from 697 to 877, with OsHAK4 being the shortest and OsHAK23 the longest (**Table 1**). The molecular weight of OsHAKs ranged from 75.03 to 94.23 kilodaltons (kDa), with OsHAK4 having the lowest and OsHAK8 the highest. Their isoelectric points ranged from 5.99 to 9.31, with OsHAK4 having the smallest and OsHAK8 the largest. All OsHAKs had positive hydrophobicity indices (0.158–0.4568), confirming their hydrophobic nature. The prediction results showed that except for OsHAK26, which was located in the nucleus, all other OsHAKs were located in the cytoplasm. NucPred scores< 0.6 for all members indicated the absence of nuclear localization signals (denoted “NA”).

**Figure 1:**
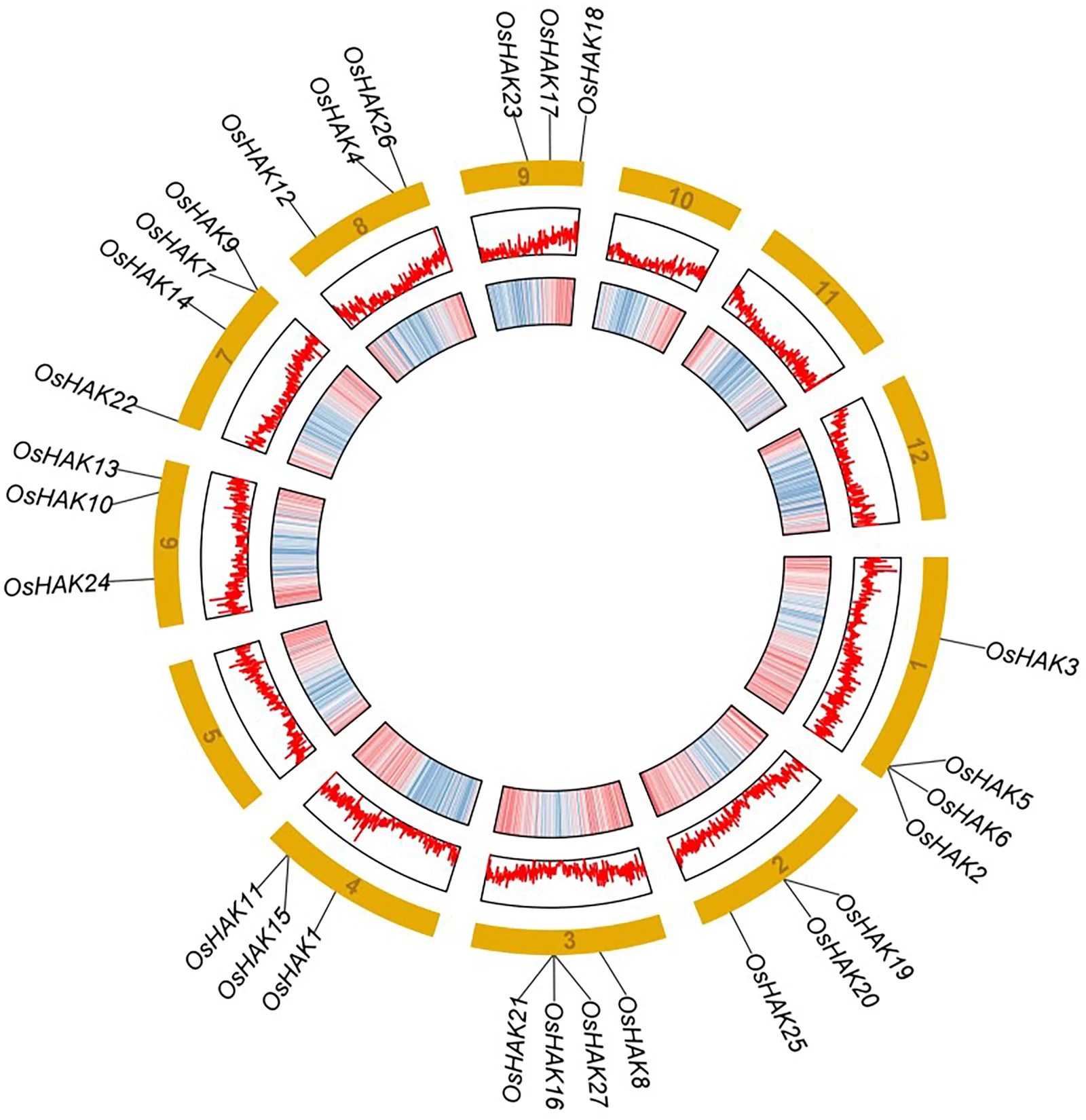
Distribution of *OsHAK* genes in rice chromosomes. Only chromosomes containing *OsHAK* genes are shown. Chromosomes are arranged circularly (1–12). Black bars indicate the location of individual OsHAK genes. The inner arc represents the gene density along the chromosome, with colors ranging from blue to red indicating increasing density.

**Table 1:** *OsHAK* genes information and sequence characteristics, predicted subcellular and nuclear localization.

Name	Gene ID	Accession number	Chromoso-me position	Length (aa)	MW (kDa)	PI	GRAVY	Localization	NLS predicts
*OsHAK1*	*Os04g0401700*	*LOC_Os04g32920*	Chr4	801	86.24	8.89	0.336	Plas	NA
*OsHAK2*	*Os01g0935500*	*LOC_Os01g70940*	Chr1	783	86.09	8.86	0.308	Plas	NA
*OsHAK3*	*Os01g0369300*	*LOC_Os01g27170*	Chr1	808	87.23	8.39	0.456	Plas	NA
*OsHAK4*	*Os08g0466200*	*LOC_Os08g36340*	Chr8	697	75.03	8.79	0.404	Plas	NA
*OsHAK5*	*Os01g0930400*	*LOC_Os01g70490*	Chr1	781	85.03	8.19	0.272	Plas	NA
*OsHAK6*	*Os01g0932500*	*LOC_Os01g70660*	Chr1	748	79.97	9.01	0.337	Plas	NA
*OsHAK7*	*Os07g0669700*	*LOC_Os07g47350*	Chr7	811	88.72	8.79	0.446	Plas	NA
*OsHAK8*	*Os03g0337500*	*LOC_Os03g21890*	Chr3	793	94.23	9.31	0.158	Plas	NA
*OsHAK9*	*Os07g0679000*	*LOC_Os07g48130*	Chr7	788	85.43	7.86	0.363	Plas	NA
*OsHAK10*	*Os06g0625900*	*LOC_Os06g42030*	Chr6	843	89.82	8.49	0.276	Plas	NA
*OsHAK11*	*Os04g0613900*	*LOC_Os04g52390*	Chr4	791	86.41	8.2	0.374	Plas	NA
*OsHAK12*	*Os08g0206400*	*LOC_Os08g10550*	Chr8	793	86.61	7.56	0.392	Plas	NA
*OsHAK13*	*Os06g0671000*	*LOC_Os06g45940*	Chr6	778	83.01	8.43	0.313	Plas	NA
*OsHAK14*	*Os07g0509200*	*LOC_Os07g32530*	Chr7	859	92.26	5.99	0.359	Plas	NA
*OsHAK15*	*Os04g0610700*	*LOC_Os04g52120*	Chr4	867	93.22	5.51	0.383	Plas	NA
*OsHAK16*	*Os03g0575200*	*LOC_Os03g37840*	Chr3	811	90.10	9.07	0.246	Plas	NA
*OsHAK17*	*Os09g0448200*	*LOC_Os09g27580*	Chr9	715	77.09	8.21	0.409	Plas	NA
*OsHAK18*	*Os09g0563200*	*LOC_Os09g38960*	Chr9	793	86.67	8.79	0.369	Plas	NA
*OsHAK19*	*Os02g0518600*	*LOC_Os02g31910*	Chr2	742	80.04	8.83	0.336	Plas	NA
*OsHAK20*	*Os02g0519100*	*LOC_Os02g31940*	Chr2	747	80.71	6.14	0.333	Plas	NA
*OsHAK21*	*Os03g0576200*	*LOC_Os03g37930*	Chr3	799	87.75	8.46	0.339	Plas	NA
*OsHAK22*	*Os07g0102100*	*LOC_Os07g01214*	Chr7	790	85.84	9.18	0.256	Plas	NA
*OsHAK23*	*Os09g0376900*	*LOC_Os09g21000*	Chr9	877	94.08	6.85	0.367	Plas	NA
*OsHAK24*	*Os06g0270200*	*LOC_Os06g15910*	Chr6	772	83.15	8.4	0.39	Plas	NA
*OsHAK25*	*Os02g0730300*	*LOC_Os02g49760*	Chr2	770	83.53	8.73	0.358	Plas	NA
*OsHAK26*	*Os08g0510300*	*LOC_Os08g39950*	Chr8	739	80.66	8.87	0.428	Nucl	NA
*OsHAK27*	*Os03g0574900*	*LOC_Os03g37830*	Chr3	811	89.66	6.77	0.225	Plas	NA

MW, Molecular weight (kDa); PI, Isoelectric point; GRAVY, Grand average of hydropathicity; NLS, Nuclear Localization Signal. Gene ID was obtained from the Ensembl Plants website (https://jun2026-plants.ensembl.org/index.html), and Accession number was sourced from the RGAP website (https://rice.uga.edu).

### HAK gene phylogenetic and gene duplication event analysis

A phylogenetic tree of HAK proteins was constructed using MEGA 11 (**Figure 2A**; **Supplementary Table 9**). The results revealed that OsHAK19 and OsHAK20 formed an evolutionarily close cluster. Furthermore, OsHAK3/4/8/10/12/15/21–23/26/27 clustered with sorghum HAK proteins, while OsHAK17 specifically aligned with BRADI4g31727V3. Conversely, OsHAK1/7/14/18 exhibited significant divergence from *B. distachyon*, sorghum, and barley orthologs, while OsHAK2/5/6/9/11/13/16/24/25 showed distant affinity to *B. distachyon* and barley lineages. MCScanX analysis identified two segmental duplications: OsHAK25 (Chr2)–OsHAK24 (Chr6) and OsHAK4 (Chr8)–OsHAK17 (Chr9) (**Figure 2B**, **Supplementary Table 3**). Notably, interspecific synteny analysis revealed conserved relationships between OsHAKs and *Arabidopsis* (1 syntenic pair), whereas 16 syntenic ortholog pairs with barley indicated significantly stronger evolutionary conservation (**Figure 2C**; **Supplementary Table 4**), suggesting preferential functional maintenance in monocot lineages.

**Figure 2:**
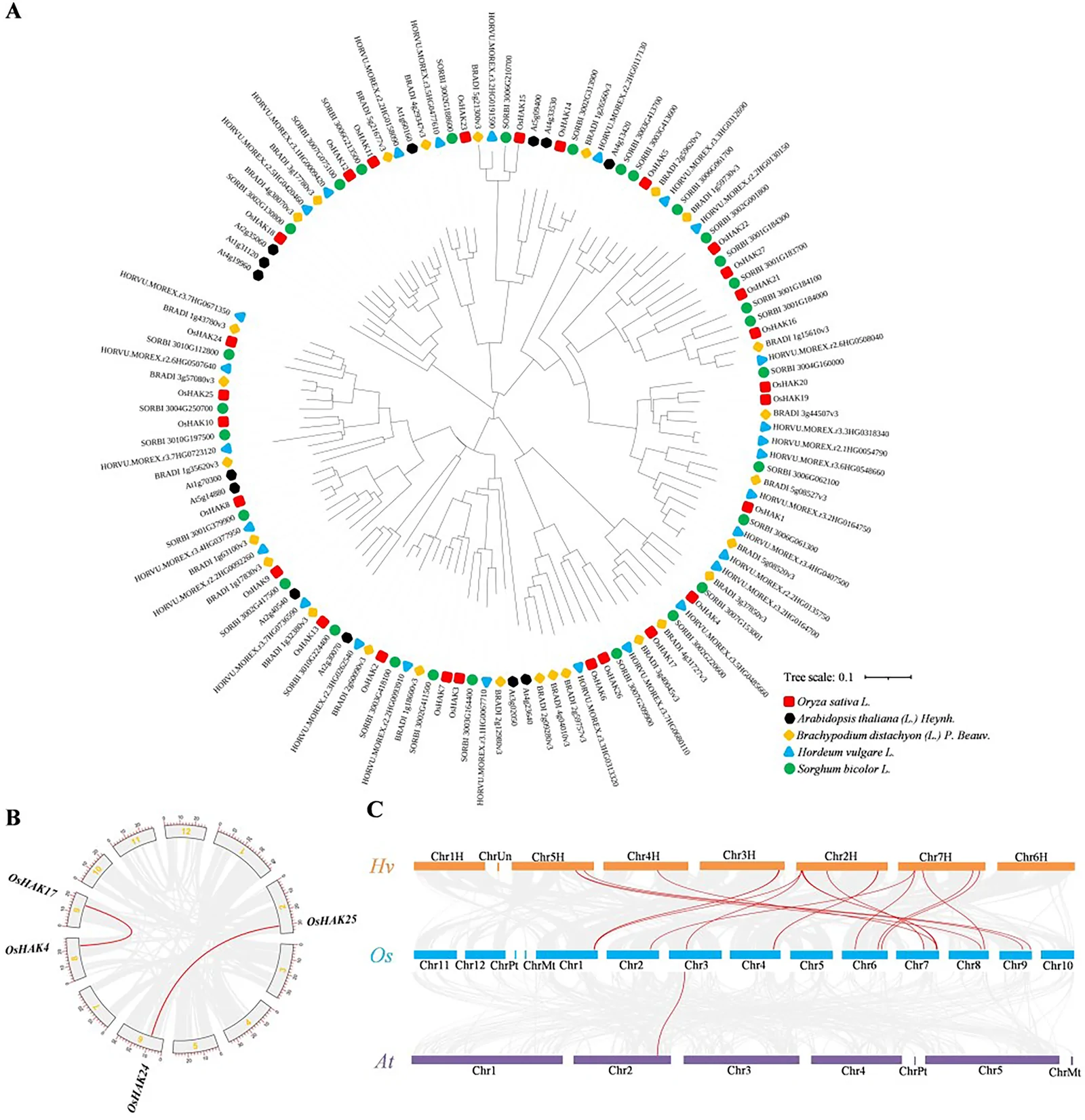
Phylogenetic tree and gene repeat analysis of the *HAK* gene family. **(A)** Phylogenetic tree depicting relationships between HAK proteins in *Oryza sativa* L. (*Os*), *Arabidopsis thaliana* (L.) Heynh. (*At*), *Brachypodium distachyon* (L.) (P) Beauv. (*BRADI*), *Hordeum vulgare* L. (*Hv*), and *Sorghum bicolor* L. (*SORBI*). **(B)** Gene segmental duplication analysis of *OsHAKs* in rice. Gray lines indicate all synteny blocks in the rice genome, and red lines represent duplicated OsHAK protein pairs. The chromosome number is indicated at the gray arc. The scale at the periphery of the chromosome represents the physical location (Kb). **(C)** Synteny analysis of *HAK* genes in the genomes between of *Oryza sativa*, *Hordeum vulgare*, and *Arabidopsis thaliana*. Gray lines show collinear blocks, while red lines indicate syntenic gene pairs between *Os* and *At* or *Hv*.

### Analysis of the gene structure, protein domains, and conserved motifs of the OsHAK gene family

Gene structure analysis of *OsHAK* members revealed lengths spanning 2.16 kb (*OsHAK3*) to 8.24 kb (*OsHAK8*), with exon numbers varying between 2 and 10 (**Figure 3A**). Notably, a significant proportion of genes contained 8–10 exons. MEME analysis identified 25 conserved motifs (Motif1–25, **Supplementary Table 4**). Conservation levels, indicated by letter height in **Figure 3B**, reflect base frequency. Except for absent motifs (e.g., Motif14/21/23 in specific OsHAKs), all members shared identical motif types, quantities, and arrangements. These structural variations likely reflect functional adaptations during evolution. Comparative sequence analysis revealed the existence of three conserved potassium-transport domains in OsHAK proteins: K_trans superfamily (PF02705), K_trans (PF08402), and Kup (PF02333) domains (**Figure 3C**). These domains are mechanistically linked to K^+^ channel function.

**Figure 3:**
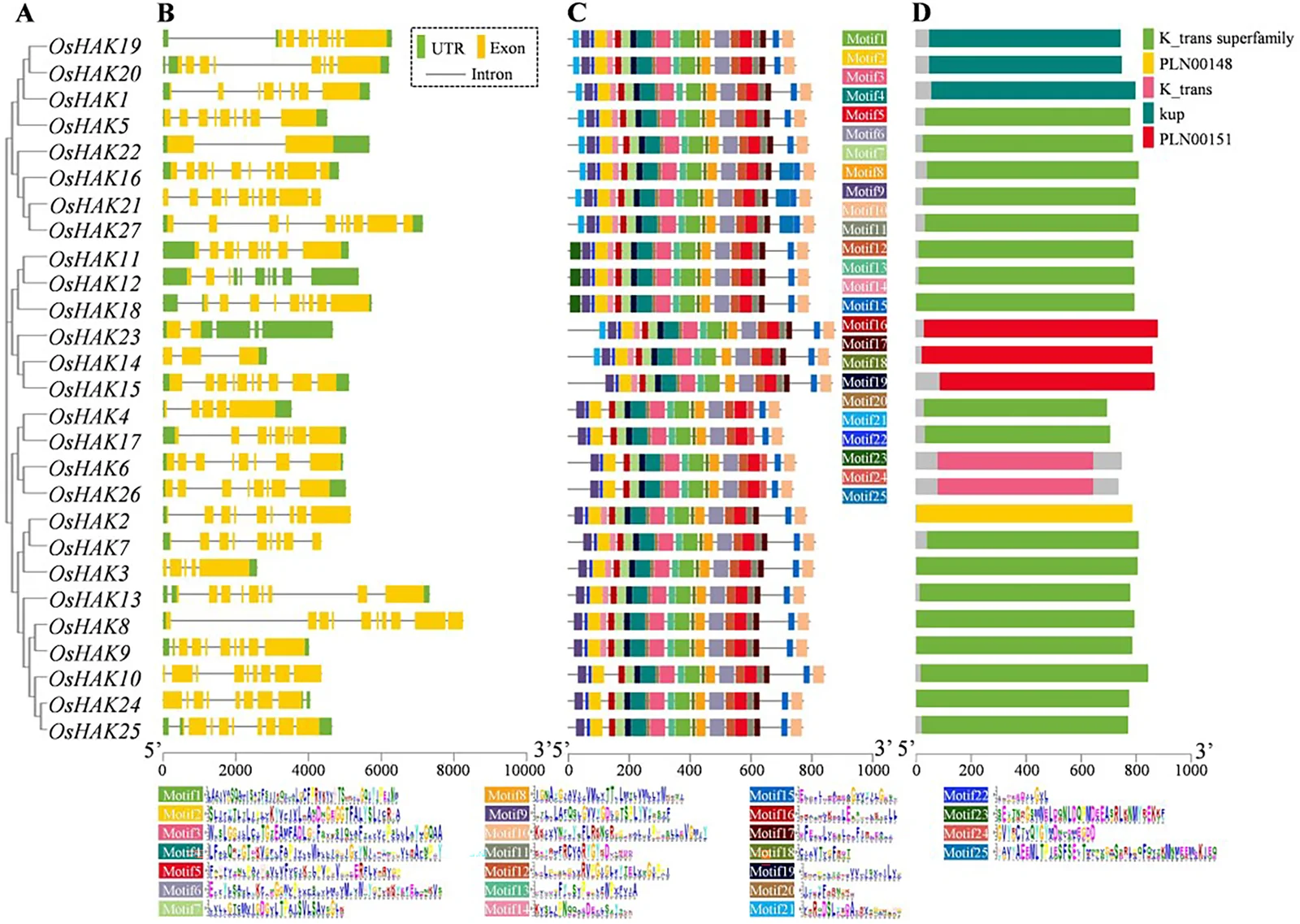
Gene structure analysis of *OsHAK* genes and conserved motif and domain analysis of OsHAK proteins. **(A)** Phylogenetic tree of OsHAK proteins. **(B)** Gene structure analysis of *OsHAK* genes. Green boxes, yellow boxes, and black lines represent UTRs, exons, and introns, respectively. **(C)** Conserved motif analysis of OsHAK proteins. **(D)** Domain distribution of OsHAK proteins.

### Analysis of protein secondary structure, tertiary structure, and transmembrane domain

The secondary structures of OsHAK proteins were predicted via SOPMA (**Table 2**). All members comprised four elements: α-helices, β-turns, random coils, and β-sheets. Typically, the proportions followed the order of α-helices > random coils > β-sheets > β-turns. Notably, random coils predominated over α-helices in OsHAK2/10/14/23. These findings imply that α-helices and random coils constitute primary structural components, whereas β-turns and β-sheets occur sporadically and may contribute to conformational flexibility.

**Table 2:** Secondary structure analysis of OsHAK proteins.

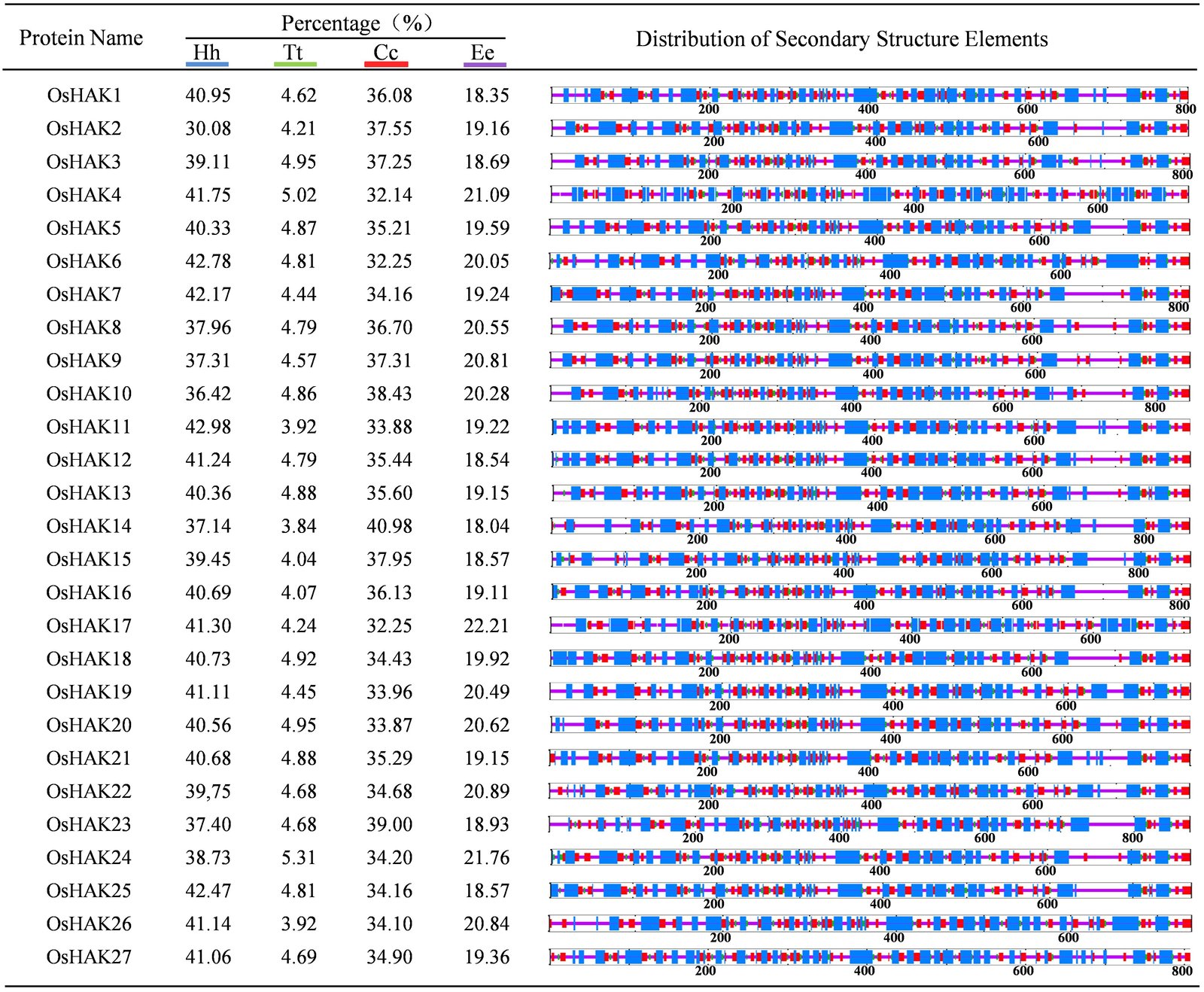

Blue represents alpha helices (Hh), green represents extended helices (Tt), yellow represents random coils (Cc), and red represents extended strands (Ee).

The tertiary structures of OsHAK proteins predicted via SWISS-MODEL exhibited conserved folding patterns (**Figure 4**). Furthermore, TMHMM2.0 analysis identified 9–13 transmembrane helices per protein, each spanning >18 residues (**Figure 5**), suggesting potential transmembrane signaling functions. Notably, 18 of 27 OsHAKs displayed N-terminal transmembrane helix probabilities >0, excluding OsHAK5/6/14–16/22–23/26–27. These N-terminal helices may function as signal peptides. Regarding subcellular orientation, cytoplasmic localization probabilities exceeded 80% for most members; however, OsHAK1 and OsHAK14 exhibited probabilities below 20%, with OsHAK14 specifically having a probability of 6.712%.

**Figure 4:**
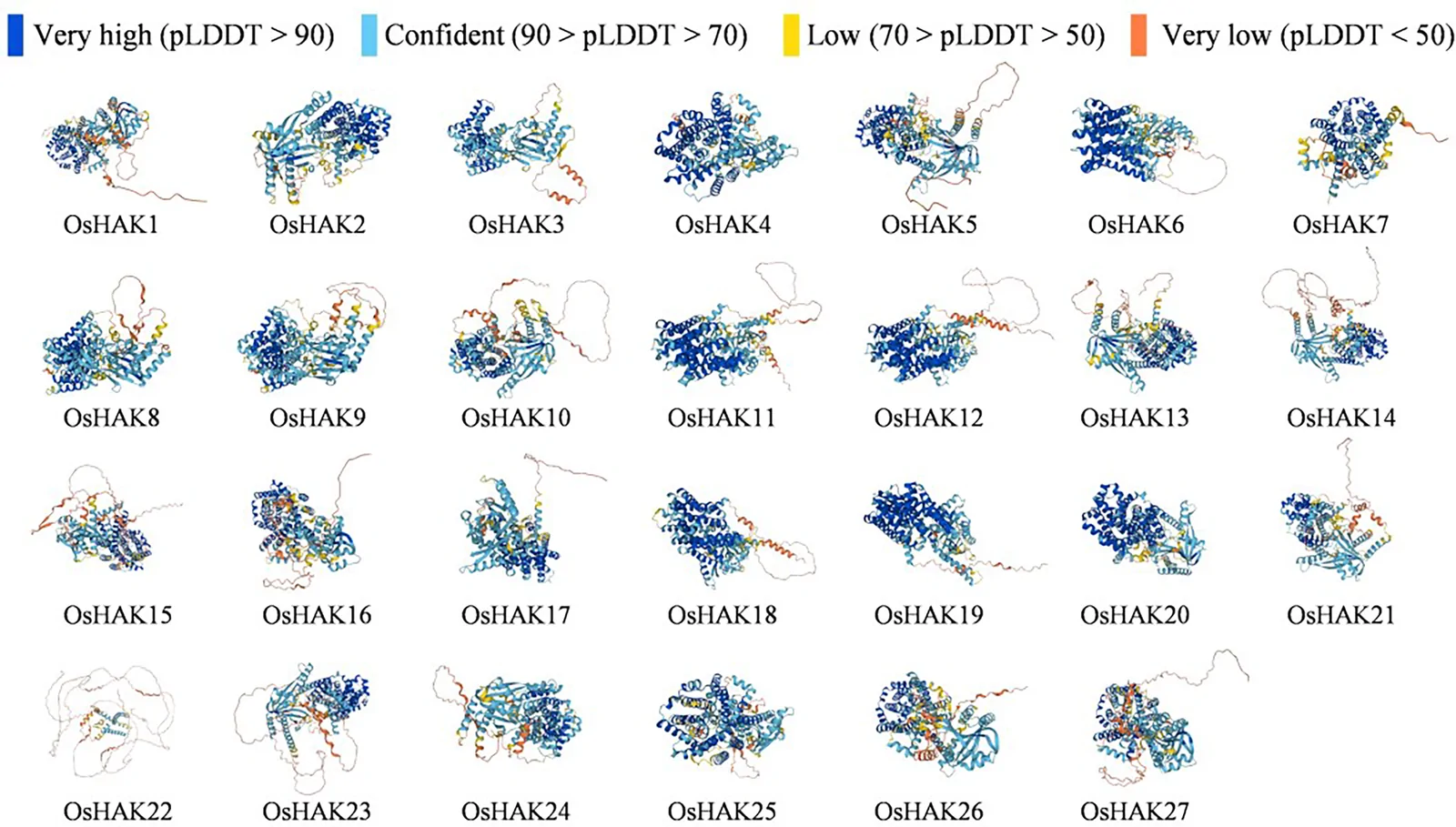
OsHAK protein three-dimensional structure prediction model. The predicted structure shows high confidence, with a per-residue predicted Local Distance Difference Test (pLDDT) score ranging from 0 to 100. A higher pLDDT value indicates higher confidence in the predicted structure.

**Figure 5:**
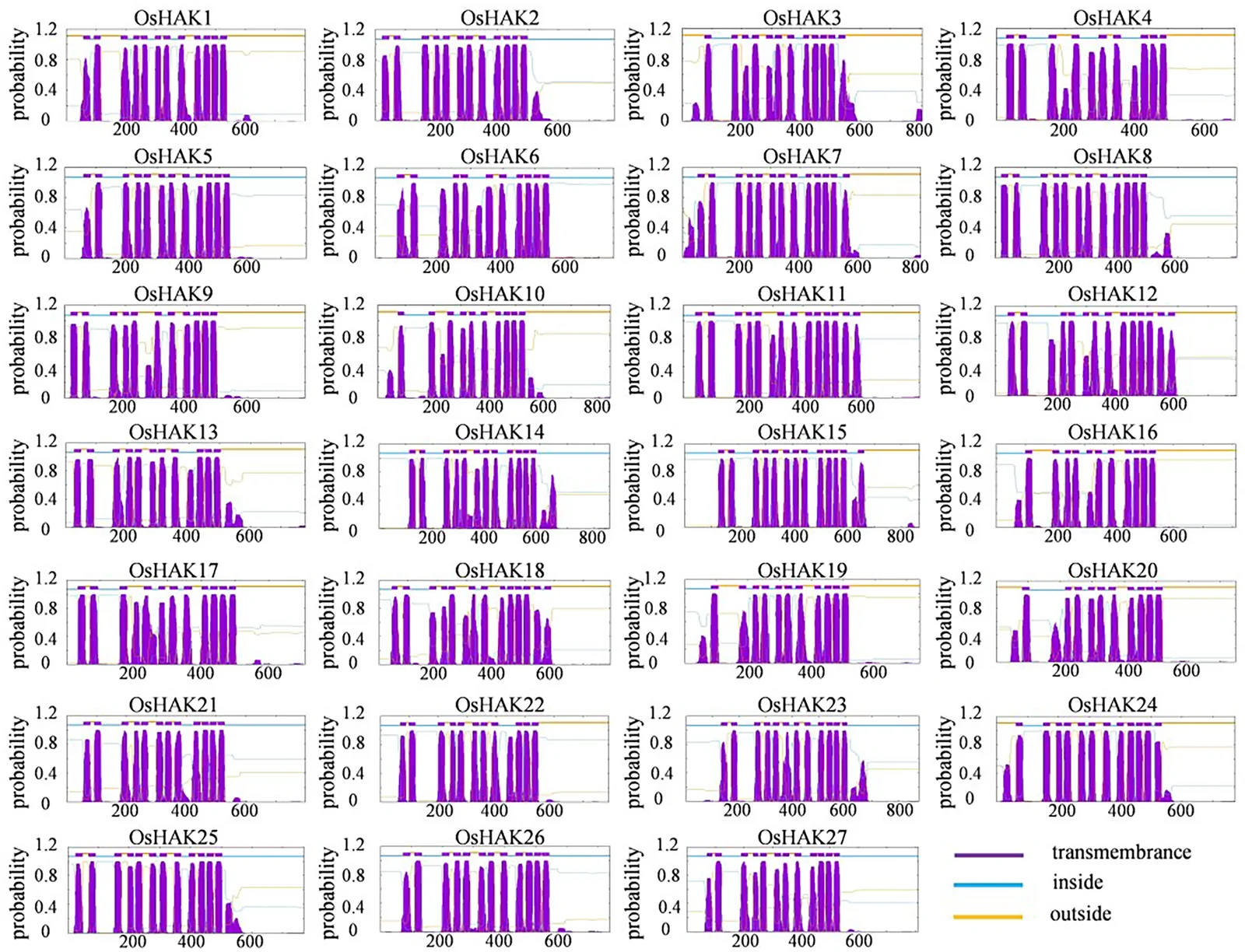
Transmembrane structure analysis of OsHAK proteins.

### OsHAK protein PPI network analysis

OsHAK protein PPI network analysis was predicted conducted using the STRING database and Cytoscape software, focusing on their potential interacting partners. As illustrated in **Figure 6**, STRING predicted that the OsHAK8, OsHAK9, OsHAK14, and OsHAK15 proteins may interact with the OsCNIH2 protein, which is classified as a cornichon protein. Additionally, the OsHAK8 protein was predicted to potentially interact with both the Os05g0461200 and Os03g0277400 proteins, while both the OsHAK11 and OsHAK18 proteins were predicted to interact with the OsEFCAX1 protein. Notably, these three members of the OsHAK family engaged in the predicted interactions with other proteins independently of most other members within this family. This suggests that various OsHAK family members may be associated with distinct physiological and biochemical pathways during rice growth and development.

**Figure 6:**
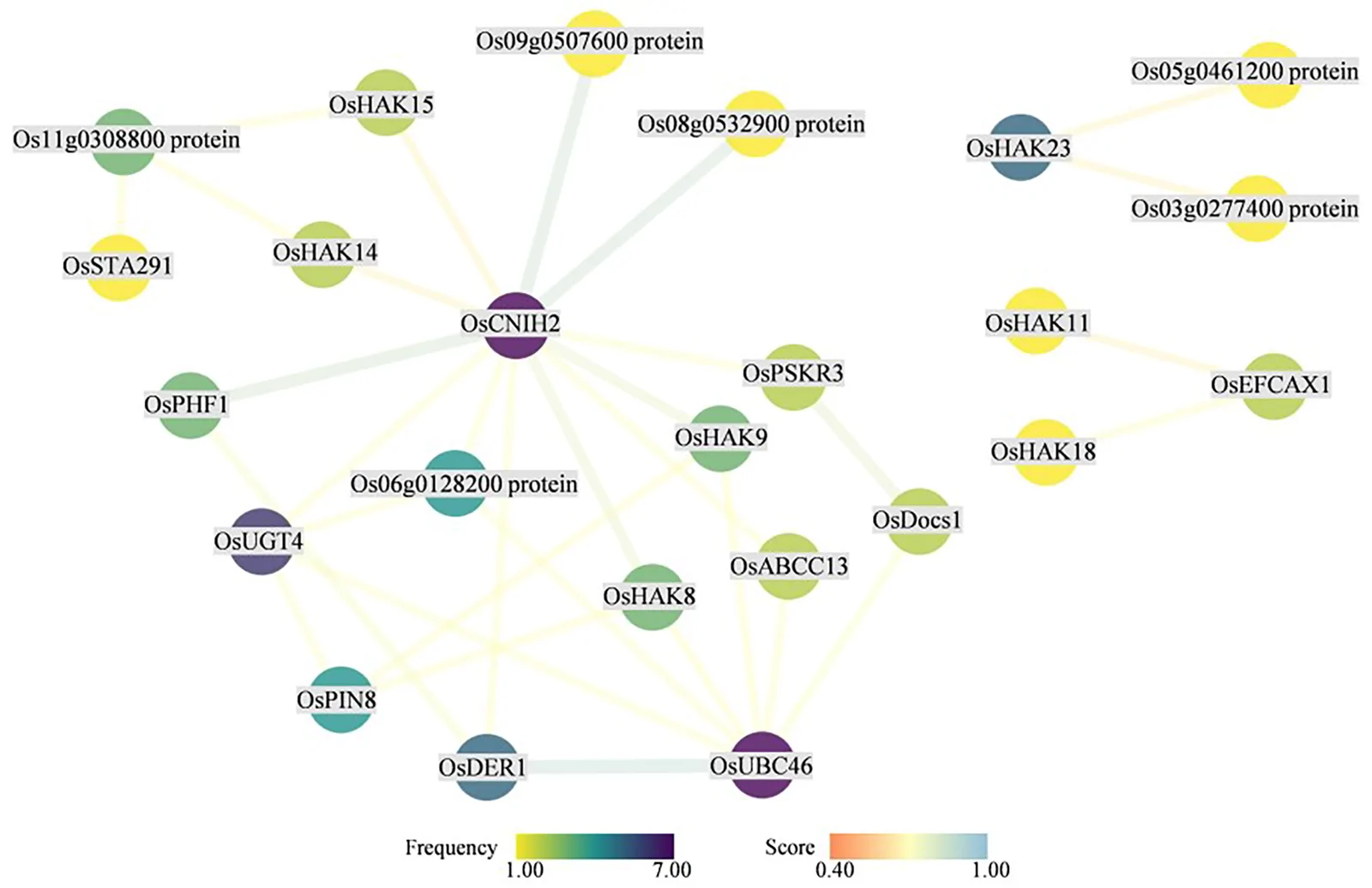
Protein–protein interaction network assembly of OsHAK proteins. The color of each circle represent the number of interacting proteins, where darker colors indicate more interacting proteins. The darker the color of the connecting circles, the higher the protein interaction score.

### Analysis of cis-acting elements in the promoter region of OsHAK family genes

Promoter *cis*-regulatory elements function as non-coding transcription factor binding sites that regulate gene expression through interactions with trans-acting factors. Analysis of -2 kb upstream sequences from *OsHAK* genes (retrieved from Ensembl) using PlantCARE revealed diverse regulatory elements (**Figure 7**; **Supplementary Tables 5, 6**), including light-responsive elements (AE-box, Box4, and G-Box), hormone-responsive elements (ABRE for abscisic acid, CGTCA/TGACG-motifs for jasmonic acid, and TCA-element for salicylic acid), stress-related elements (AP-1, MYB, MYC, STRE, and TC-rich repeats), and developmental elements (CAT-box). These findings indicate that *OsHAK* genes integrate light, hormonal, and environmental signals to coordinate growth–stress responses. Notably, an AP-1 element responsive to cadmium stress occurred exclusively in OsHAK3, while GATT-motif and ATC-motif light-response elements displayed reduced frequency across the family.

**Figure 7:**
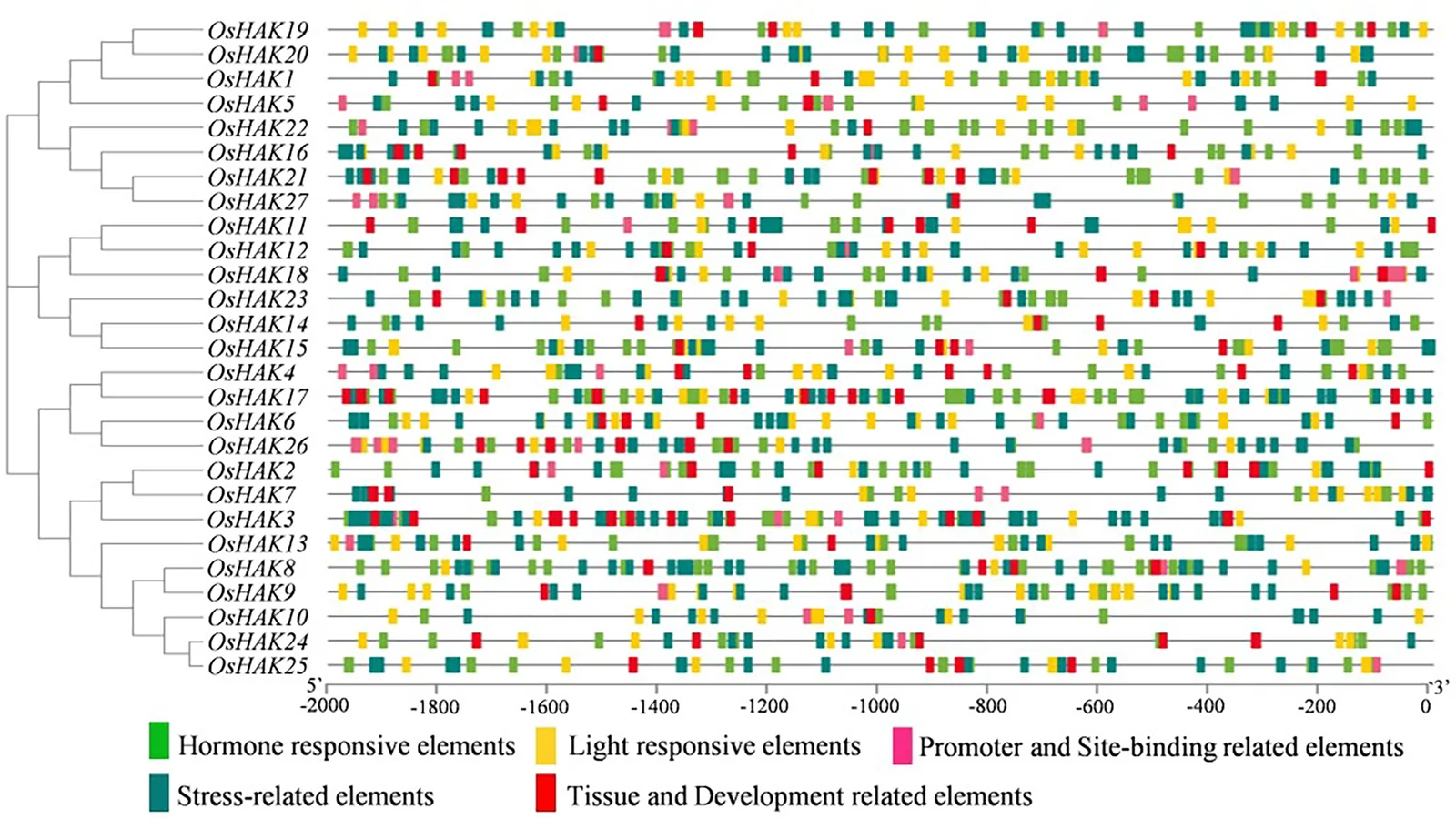
Predicted *cis*-acting elements in *OsHAK* promoter regions. Promoter sequences (-2 kb) of 27 *OsHAKs* were analyzed using PlantCARE. Different colors indicate different *cis*-elements.

### Analysis of the expression profile of the OsHAK family and the expression levels of its family members under salt treatment conditions

Expression profile of *OsHAK* in various rice organs and developmental stages (RGAP data, **Figure 8A**; **Supplementary Table 7**) revealed distinct tissue-specific expression. To highlight the differences between tissues, the data were processed using log2 transformation. *OsHAK1* and *OsHAK7* were highly expressed in roots and stems, *OsHAK8* exhibited the highest expression in young panicles, and *OsHAK27* showed the highest expression in leaves. Concurrently, *OsHAK3*, *OsHAK6*, *OsHAK13*, and *OsHAK20* maintained constitutively low transcription levels. Collectively, these results indicated that the expression of *OsHAK* family genes displayed distinct patterns across different tissues and developmental stages. qRT-PCR analysis of salt stress responses (**Figure 8B**) identified significant downregulation in *OsHAK2* (shoots), *OsHAK13*/*19* (shoots), and *OsHAK23* (roots). Conversely, *OsHAK1*, *OsHAK8*, *OsHAK11*, *OsHAK14*, *OsHAK15*, *OsHAK23* (shoots), *OsHAK24*, *OsHAK25*, and *OsHAK27* were upregulated. These differential expression patterns indicated both tissue-specific functional diversification and specialized roles in ionic stress adaptation mechanisms.

**Figure 8:**
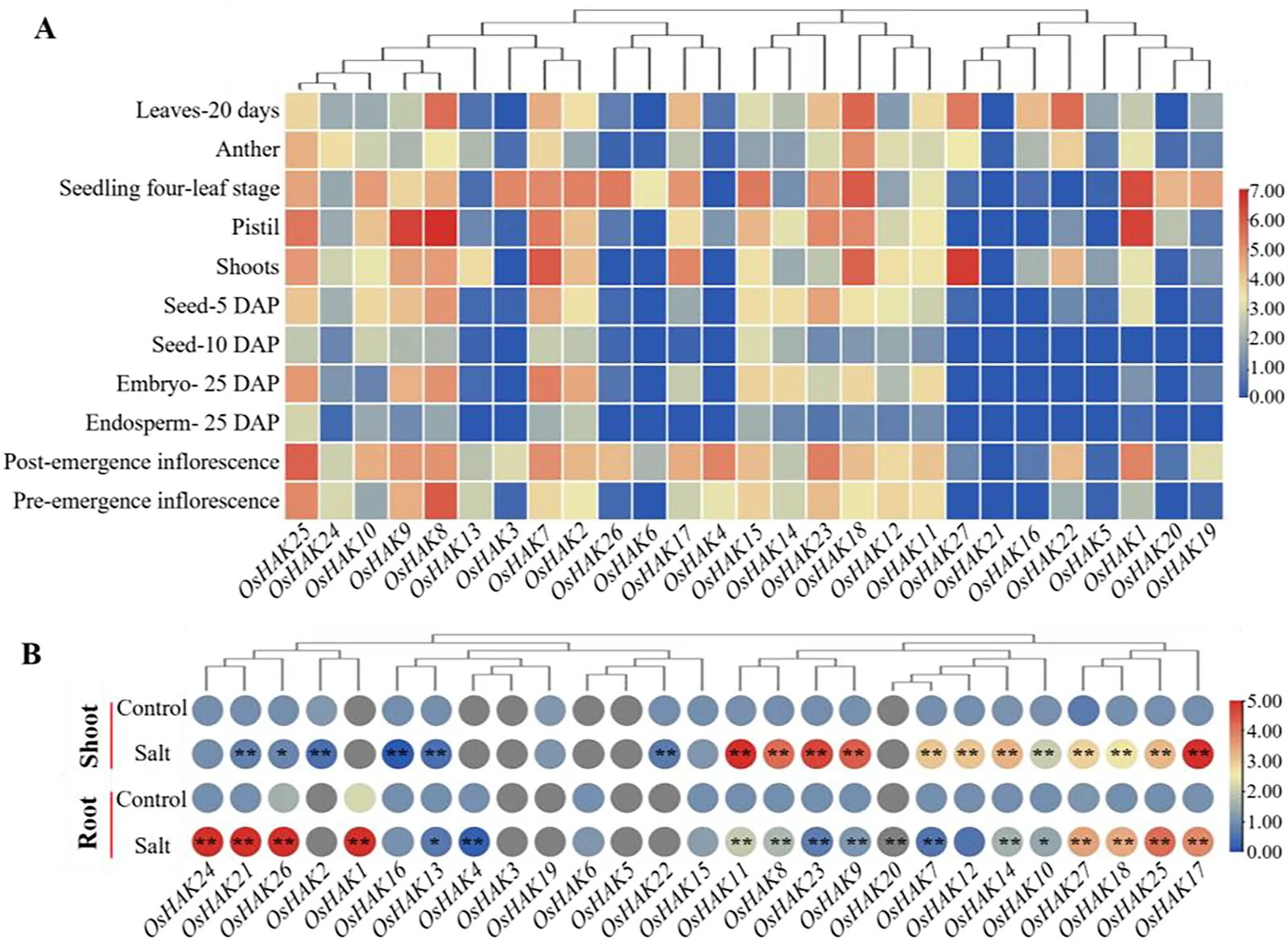
Tissue-specific expression profiling of *OsHAKs* and their responsiveness to salt stress. **(A)** Expression profiles of *OsHAKs* in the *Oryza sativa* (L) spp. *japonica* obtained from the RGAP database. The color scale represents relative expression levels from low (blue) to high (red). **(B)** Expression analysis of *OsHAKs* under 140 mM NaCl treatment for 5 h. Total RNA was extracted from the shoots of 14-day-old rice seedlings under both induced salt stress and no salt stress. The color scale represents relative expression levels from low (blue) to high (red). Data are presented as the mean ± SD from three independent biological replicates. Statistical significance was determined by Student’s t-test (*P ≤ 0.05; **P ≤ 0.01).

### Functional analysis of some members of the OsHAK family in the K+ uptake-defective yeast strain CY162

The functional characterization of unreported *OsHAK11*, *OsHAK13*, and *OsHAK23–26* potassium transporters was performed through yeast complementation assays. Full-length CDSs (**Supplementary Table 8**) were cloned into pYES2 and transformed into the K^+^-uptake-deficient yeast strain CY162. As potassium concentrations declined in AP medium (**Figure 9**), *AtKAT1*-transformed strains (positive control) maintained growth, while empty vector controls failed at ≤1 mM K^+^. Strains expressing *OsHAK11* and *OsHAK25* exhibited partial functional complementation, surviving at 0.5 mM K^+^, although reduced growth was observed compared with *AtKAT1*. Other tested *OsHAKs* (*OsHAK13/23/24/26*) displayed minimal growth under equivalent conditions. These results demonstrate differential complementation capacity, with *OsHAK11* and *OsHAK25* providing superior restoration of K^+^ uptake deficiency relative to other family members.

**Figure 9:**
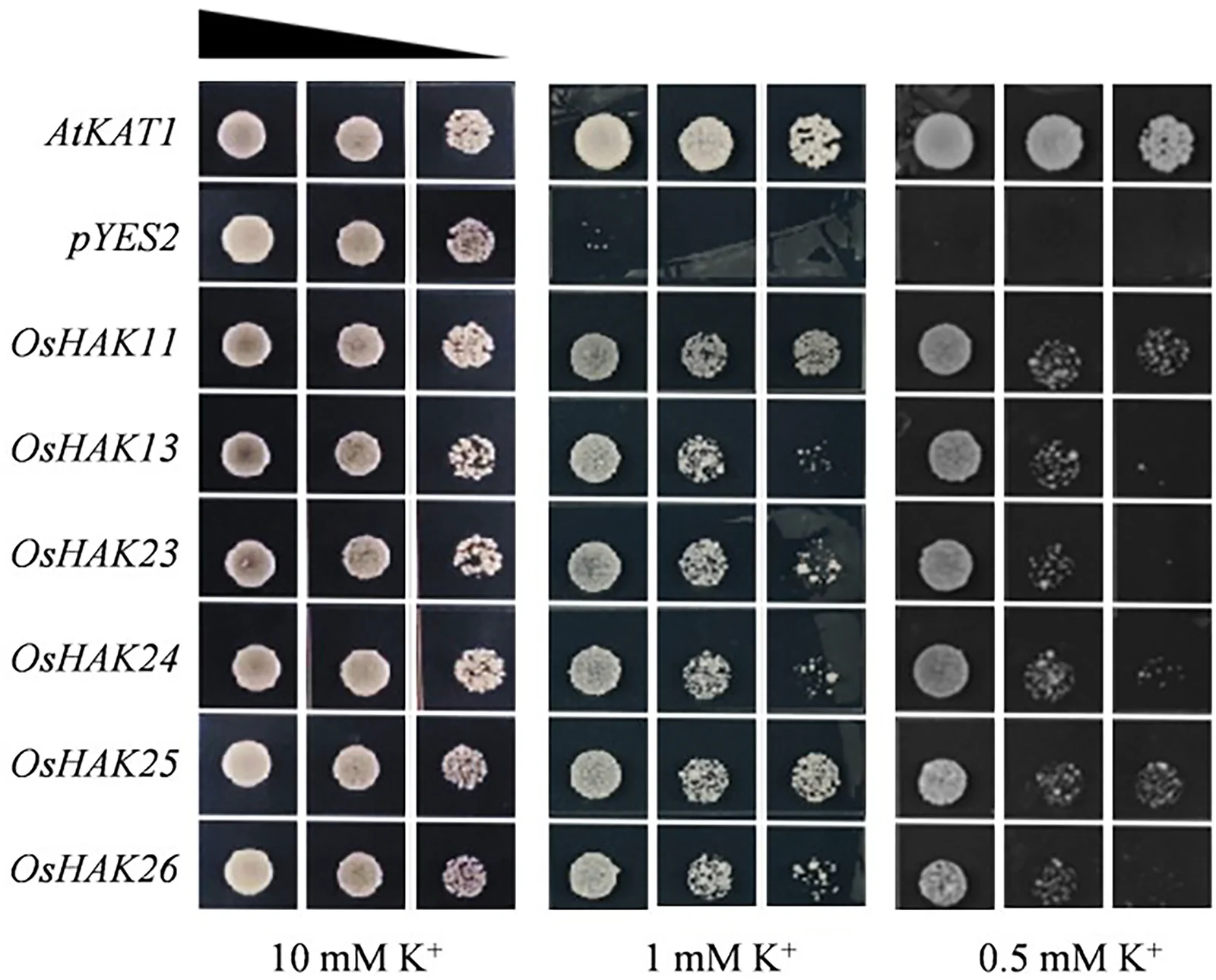
*OsHAKs* rescued the deficiency of potassium uptake in CY162. Growth statuses of CY162 cells expressing *AtKAT1*, *OsHAK11*, *OsHAK13*, *OsHAK23*, *OsHAK24*, *OsHAK25*, *OsHAK26*, and *pYES2* on AP medium with different potassium concentrations. The black triangle indicates a 10-fold gradient dilution, and 5 μl of each concentration was inoculated on the medium.

### OsHAK11 and OsHAK25 localization analysis

To validate protein subcellular localization, we generated fusion constructs of plasma membrane marker *AtCBL1-mCherry* with *OsHAK11-cGFP* and *OsHAK25-cGFP*. These constructs were transiently expressed in tobacco leaves. Confocal microscopy revealed diffuse cGFP fluorescence (empty vector control) throughout the nucleus, cytoplasm, and plasma membrane. In contrast, co-expression assays demonstrated the precise co-localization of *OsHAK11-cGFP*/*AtCBL1-mCherry* and *OsHAK25-cGFP*/*AtCBL1-mCherry* at the cell periphery, as evidenced by overlapping fluorescence signals (**Figure 10**). These results confirmed the plasma membrane targeting of both OsHAK11 and OsHAK25, in accordance with computational predictions.

**Figure 10:**
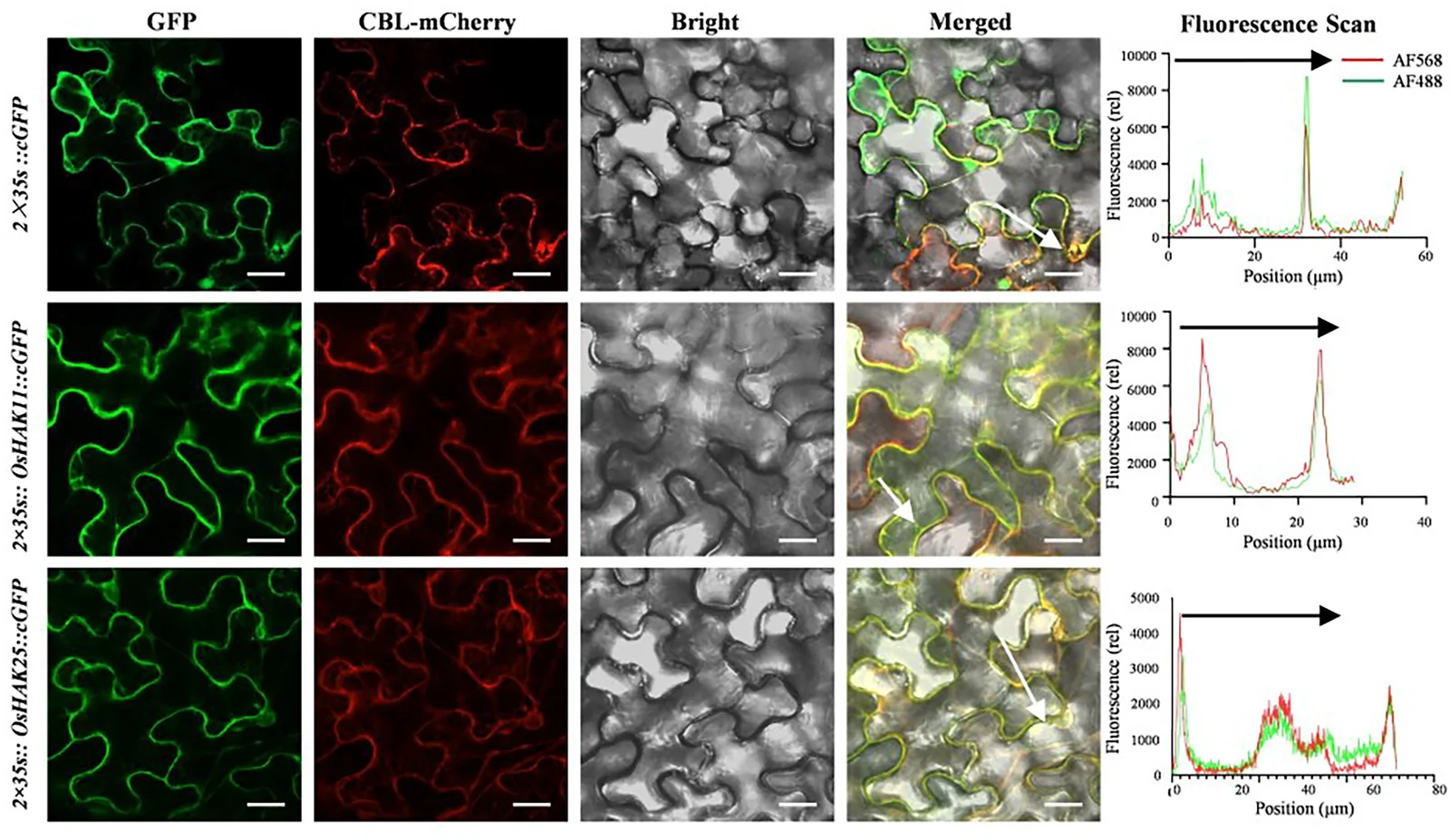
Subcellular localization of OsHAK11 and OsHAK25 in tobacco. *2×35s::OsHAK11-cGFP*, *2×35s::OsHAK25-cGFP*, and localization signal-tagged mCherry were co-transformed into tobacco leaf cells. *2×35s::cGFP* converted to control. Scale bar = 40 μm. *CBL-mCherry* indicates plasma membrane localization signal-tagged mCherry.

## Discussion

### Characteristics and evolution of the OsHAK family

The *HAK* family represents the most prevalent and significant K^+^ transporter protein family and is extensively distributed across bacteria, fungi, and plants (Corratgé-Faillie et al., 2010). Previous research has identified 30 members of this family in quinoa (Chen et al., 2023b), 27 in barley (Cai et al., 2021), 56 in wheat (Cheng et al., 2018), and 31 in poplar (He et al., 2012). In this study, we investigated 27 members of the rice *HAK* family. Notably, there appears to be a correlation between genetic proximity to rice and the number of *HAK* family members; this observation suggests that *HAK* family members exhibit a degree of evolutionary conservation.

Phylogenetic analysis of multiple species has revealed a relatively close relationship between *OsHAK5* and *AtHAK5* (*At4G13420*). Furthermore, research indicates that both *OsHAK5* and *AtHAK5* play significant roles in K^+^ uptake under conditions of limited K^+^ availability (Genies et al., 2021; Uchiyama et al., 2022; Maierhofer et al., 2024), suggesting that the phylogenetic analysis presented in **Figure 3A** is relatively accurate. Segmental duplication serves as the primary driving force underlying the expansion of the KT/HAK/KUP family and is subject to purifying selection (Ka/Ks< 1) during domestication. This finding aligns with the results of previous intraspecific collinearity analysis conducted on *HAK* family members across watermelon, *Ipomoea*, and cotton (Jin et al., 2021; Azeem et al., 2022; Cheng et al., 2024).

Transmembrane domains mainly consist of α-helical segments of amino acids that exhibit hydrophobic characteristics, allowing them to integrate into the hydrophobic core of the cell membrane. This structural feature facilitates various functions, including the transport of substances between intracellular and extracellular environments as well as the regulation of transmembrane signal transduction. The data presented in **Table 2** indicate that the secondary structure of rice HAK proteins is primarily α-helical. Furthermore, the prediction results depicted in **Figure 5** indicate that each OsHAK protein encompasses multiple transmembrane domains, reinforcing the notion that OsHAK family proteins possess a structural foundation for ion transport or for transporting substances such as K^+^ and Na^+^ (Azeem et al., 2022; Guo et al., 2024). Additionally, research has demonstrated that *Cajanus cajan* and Chinese cabbage contain diverse *cis*-regulatory elements associated with abiotic stress responses. This finding aligns with *cis*-element prediction results observed in rice (**Figure 7**) (Shen and Yuan, 2021; Siddique et al., 2021). It is hypothesized that members of the *OsHAK* gene family respond to abiotic stress by engaging in substance transport or signal transduction.

Protein–protein interaction prediction showed that OsHAK8, OsHAK9, OsHAK14, and OsHAK15 interact with OsCNIH2, whereas OsHAK11 and OsHAK18 interact with OsEFCAX1 (**Figure 6**). The CNIH family comprises conserved COPII vesicle cargo receptors that facilitate the export of membrane proteins from the endoplasmic reticulum and their delivery to target membranes via the secretory pathway (Kacovská et al., 2026). OsCNIH1 has been reported to interact with OsHKT1;3, guiding the Na^+^ transporter to the Golgi apparatus, and OsCNIH2 is highly homologous to OsCNIH1 (Rosas-Santiago et al., 2015). These findings suggest that the localization and sorting of OsHAK8, OsHAK9, OsHAK14, and OsHAK15 may be regulated by OsCNIH2. OsEFCAX1 is an EF-hand-containing H^+^/cation exchanger (Singh et al., 2014). The EF-hand domain is a canonical Ca²^+^-binding motif. This implies that OsHAK11 and OsHAK18 may be subject to Ca²^+^-dependent regulatory pathways that affect K^+^ transport.

### Response of the OsHAK family to salt stress

The data indicate that *OsHAK5* and *OsHAK1* encode transporters facilitating K^+^ acquisition under salt stress. OsHAK5 mediates selective K^+^ uptake with minimal Na^+^ interference, while OsHAK1, induced by salinity, enhances salt tolerance by maintaining K^+^/Na^+^ homeostasis and scavenging reactive oxygen species (Horie et al., 2011; Yang et al., 2014; Chen et al., 2015). Consistent with these findings, OsHAK1, which is responsible for K^+^ uptake, has been reported to be significantly induced by salt stress at the transcriptional level (Chen et al., 2015). Accordingly, our results also showed that OsHAK1 was significantly induced by salt stress in rice roots. Furthermore, OsHAK21 forms a complex with OsCYB5–2 to regulate the salt tolerance of rice (Song et al., 2021). *OsCYB5–2* enhances the apparent affinity of OsHAK21 for K^+^ binding and increases its transport activity. Concurrently, high salinity directly stimulates the interaction between OsHAK21 and OsCYB5-2, thereby promoting OsHAK21-mediated K^+^ uptake while restricting Na^+^ entry into cells, thus preserving intracellular K^+^/Na^+^ homeostasis (Song et al., 2021). Consistent with these findings, our results showed that OsHAK21 expression was significantly upregulated in roots under salt stress (Shen et al., 2015). Under salt stress, the expression level of OsHAK4 in the underground portion of seedlings declines markedly (Che et al., 2024). Under salt treatment conditions, the expression level of *OsHAK7* in aboveground plant tissues is significantly elevated, while its expression in the underground parts of plants is notably reduced. Conversely, the expression level of *OsHAK10* significantly increases. Under salt stress, the expression level of *OsHAK18* is significantly elevated (Shen et al., 2023). In this study, OsHAK11 and OsHAK25 were upregulated under salt stress, whereas OsHAK13 was downregulated, despite all three transporters exhibiting K^+^ transport activity in yeast. This discrepancy may be attributed to differences in their promoter cis-elements. OsHAK11 uniquely contains a TGA-box and DRE1, while OsHAK25 uniquely harbors a circadian element. DRE1 is recognized by DREB/CBF transcription factors, which are core regulators of abiotic stress tolerance, whereas the TGA-box integrates salicylic acid and auxin signaling. The unique presence of these two elements in OsHAK11 suggests that its upregulation under salt stress may be mediated through the DREB/CBF pathway. The circadian element in OsHAK25 implies that its expression may be regulated by the circadian clock, potentially contributing to the temporal control of K^+^ uptake under stress. In contrast, OsHAK13 contains a W box and WRE3 but lacks the TGA-box, DRE1, and circadian element, which may partially explain why OsHAK13 is downregulated under salt stress despite its K^+^ transport activity in yeast.

### Response of the OsHAK family to low potassium

Yeast functional complementation analysis has demonstrated that SsHAK1, SsHAK21, TaHAK13, and TaHAK18 can restore the K^+^ uptake capacity of yeast mutants (Feng et al., 2020; Cheng et al., 2024; Liu et al., 2025). Additionally, research has shown that the expression of HbHAK1 in the yeast strain CY162 results in strong K^+^ uptake activity under extremely low external K^+^ conditions and reduces Na^+^ toxicity to maintain cell survival under high salinity stress (Zhang et al., 2020a). These findings suggest that multiple HAK family proteins possess K^+^ transport capabilities.

In this study, the functional analysis results for certain members of the OsHAK family in the K^+^ absorption-deficient yeast strain CY162 suggested that OsHAK11, OsHAK13, OsHAK23, OsHAK24, OsHAK25, and OsHAK26 exhibited functions similar to AtKAT1 in mediating K^+^ uptake in yeast cells (**Figure 9**). Notably, among the tested OsHAKs, both OsHAK11 and OsHAK25 demonstrated a relatively strong ability to complement potassium absorption deficiency in yeast. This may be attributed to the N-terminal transmembrane helix probability of OsHAK11 and OsHAK25 being greater than zero, which may function as a signal peptide or affect membrane insertion efficiency, thereby influencing transport activity. Furthermore, OsHAK25 and OsHAK24 constitute a segmental duplication pair; however, only OsHAK25 exhibited strong complementation ability, suggesting that this duplication pair has undergone functional divergence after duplication rather than simply retaining redundant functions.Genome-wide association studies and quantitative trait locus mapping have implied that among these two proteins, OsHAK25 may be useful for enhancing salt tolerance in rice (Nayyeripasand et al., 2021). However, there is currently no experimental evidence explicitly demonstrating that HAK25 can improve salt tolerance. The K^+^ uptake capacity of HAK proteins does not necessarily correlate with their functions under salt stress, as salt tolerance in plants is primarily determined by the intracellular Na^+^/K^+^ ratio rather than the absolute rate of K^+^ uptake. Therefore, potassium uptake complementation assays alone are insufficient to directly demonstrate the salt tolerance function of HAK members. Additional evidence from yeast salt tolerance assays, such as measuring yeast growth phenotypes in media containing different Na^+^ concentrations, is required to further validate their roles in salt stress responses.

## Conclusions

The HAK family of potassium transport proteins plays a crucial role in the plant potassium transport system and salt tolerance. In this study, we conducted a systematic bioinformatics analysis of the *HAK* gene family in rice. Our predictions indicated that all 27 OsHAK proteins possess transmembrane domains and that their upstream regions contain *cis*-acting elements that respond to abiotic stress. Expression pattern analysis revealed functional differences among OsHAK proteins regarding K^+^ absorption and response to salt stress. Notably, under low K^+^ conditions, compared with other tested OsHAKs, *OsHAK11* and *OsHAK25* exhibited a relatively strong ability to complement yeast K^+^ deficiencies, suggesting they may play key roles in maintaining Na^+^/K^+^ balance. The subcellular localization results showed that both *OsHAK11* and *OsHAK25* are localized at the plasma membrane, consistent with predictions. This study not only enhances our understanding of the classification of the *OsHAK* gene family as well as its protein physicochemical properties and structures but also provides valuable insights supporting further exploration into the mechanisms by which these genes respond to salt stress.

## Data Availability

The datasets presented in this study can be found in online repositories. The names of the repository/repositories and accession number(s) can be found in the article/**Supplementary Material.**

## References

[B1] Anderson J. A. Huprikar S. S. Kochian L. V. Lucas W. J. Gaber R. F. (1992). Functional expression of a probable Arabidopsis thaliana potassium channel in Saccharomyces cerevisiae. Proc. Natl. Acad. Sci. U.S.A. 89, 3736–3740. doi: 10.1073/pnas.89.9.3736 1570292 PMC525565

[B2] Azeem F. Zameer R. Rehman Rashid M. A. Rasul I. Ul-Allah S. Siddique M. H. . (2022). Genome-wide analysis of potassium transport genes in Gossypium raimondii suggest a role of GrHAK/KUP/KT8, GrAKT2.1 and GrAKT1.1 in response to abiotic stress. Plant Physiol. Biochem. 170, 110–122. doi: 10.1016/j.plaphy.2021.11.038 34864561

[B3] Cai K. Zeng F. Wang J. Zhang G. (2021). Identification and characterization of HAK/KUP/KT potassium transporter gene family in barley and their expression under abiotic stress. BMC Genomics 22, 317. doi: 10.1186/s12864-021-07633-y 33932999 PMC8088664

[B7] Chen G. Hu Q. Luo L. Yang T. Zhang S. Hu Y. . (2015). Rice potassium transporter OsHAK1 is essential for maintaining potassium-mediated growth and functions in salt tolerance over low and high potassium concentration ranges. Plant Cell Environ. 38, 2747–2765. doi: 10.1111/pce.12585 26046301

[B5] Chen C. Wu Y. Li J. Wang X. Zeng Z. Xu J. . (2023a). TBtools-II: A "one for all, all for one" bioinformatics platform for biological big-data mining. Mol. Plant 16, 1733–1742. doi: 10.1016/j.molp.2023.09.010 37740491

[B8] Chen Y. Lin Y. Zhang S. Lin Z. Chen S. Wang Z. (2023b). Genome-wide identification and characterization of the HAK gene family in Quinoa (Chenopodium quinoa Willd.) and their expression profiles under saline and alkaline conditions. Plants (Basel) 12. doi: 10.3390/plants12213747 37960103 PMC10650088

[B4] Che J. Yamaji N. Wang S. F. Xia Y. Yang S. Y. Su Y. H. . (2024). OsHAK4 functions in retrieving sodium from the phloem at the reproductive stage of rice. Plant J. 120, 76–90. doi: 10.1111/tpj.16971 39139125

[B6] Chen D. Tian Q. Wu Q. Cao H. Ding K. Jing W. . (2026). A brassinosteroid signaling protein acts as an integrative regulator of K+ and Na+ transport in rice response to salt stress. Plant Cell. 38 (9), koag274. doi: 10.1093/plcell/koag274 42725881

[B10] Cheng X. Liu X. Mao W. Zhang X. Chen S. Zhan K. . (2018). Genome-wide identification and analysis of HAK/KUP/KT potassium transporters gene family in wheat (Triticum aestivum L.). Int. J. Mol. Sci. 19. doi: 10.3390/ijms19123969 30544665 PMC6321448

[B9] Cheng R. Zhao Z. Tang Y. Gu Y. Chen G. Sun Y. . (2024). Genome-wide survey of KT/HAK/KUP genes in the genus Citrullus and analysis of their involvement in K^+^-deficiency and drought stress responses in between C. lanatus and C. amarus. BMC Genomics 25, 836. doi: 10.1186/s12864-024-10712-5 39237905 PMC11378637

[B11] Corratgé-Faillie C. Jabnoune M. Zimmermann S. Véry A. A. Fizames C. Sentenac H. (2010). Potassium and sodium transport in non-animal cells: the Trk/Ktr/HKT transporter family. Cell. Mol. Life Sci. 67, 2511–2532. doi: 10.1007/s00018-010-0317-7 20333436 PMC11115768

[B12] Feng X. Wang Y. Zhang N. Wu Z. Zeng Q. Wu J. . (2020). Genome-wide systematic characterization of the HAK/KUP/KT gene family and its expression profile during plant growth and in response to low-K^+^ stress in Saccharum. BMC Plant Biol. 20, 20. doi: 10.1186/s12870-019-2227-7 31931714 PMC6958797

[B13] Genies L. Martin L. Kanno S. Chiarenza S. Carasco L. Camilleri V. . (2021). Disruption of AtHAK/KT/KUP9 enhances plant cesium accumulation under low potassium supply. Physiol. Plant 173, 1230–1243. doi: 10.1111/ppl.13518 34342899

[B14] Gierth M. Mäser P. Schroeder J. I. (2005). The potassium transporter AtHAK5 functions in K(+) deprivation-induced high-affinity K(+) uptake and AKT1 K(+) channel contribution to K(+) uptake kinetics in Arabidopsis roots. Plant Physiol. 137, 1105–1114. doi: 10.1104/pp.104.057216 15734909 PMC1065410

[B15] Guo H. Tan J. Jiao Y. Huang B. Ma R. Ramakrishnan M. . (2024). Genome-wide identification and expression analysis of the HAK/KUP/KT gene family in Moso bamboo. Front. Plant Sci. 15, 1331710. doi: 10.3389/fpls.2024.1331710 38595761 PMC11002169

[B16] Han M. Wu W. Wu W. H. Wang Y. (2016). Potassium transporter KUP7 is involved in K(+) acquisition and translocation in Arabidopsis root under K(+)-limited conditions. Mol. Plant 9, 437–446. doi: 10.1016/j.molp.2016.01.012 26851373

[B17] He C. Cui K. Duan A. Zeng Y. Zhang J. (2012). Genome-wide and molecular evolution analysis of the Poplar KT/HAK/KUP potassium transporter gene family. Ecol. Evol. 2, 1996–2004. doi: 10.1002/ece3.299 22957200 PMC3434002

[B18] He Y. Yang B. He Y. Zhan C. Cheng Y. Zhang J. . (2019). A quantitative trait locus, qSE3, promotes seed germination and seedling establishment under salinity stress in rice. Plant J. 97, 1089–1104. doi: 10.1111/tpj.14181 30537381 PMC6850641

[B19] Hong J. P. Takeshi Y. Kondou Y. Schachtman D. P. Matsui M. Shin R. (2013). Identification and characterization of transcription factors regulating Arabidopsis HAK5. Plant Cell Physiol. 54, 1478–1490. doi: 10.1093/pcp/pct094 23825216

[B20] Horie T. Sugawara M. Okada T. Taira K. Kaothien-Nakayama P. Katsuhara M. . (2011). Rice sodium-insensitive potassium transporter, OsHAK5, confers increased salt tolerance in tobacco BY2 cells. J. Biosci. Bioeng. 111, 346–356. doi: 10.1016/j.jbiosc.2010.10.014 21084222

[B21] Jin R. Jiang W. Yan M. Zhang A. Liu M. Zhao P. . (2021). Genome-wide characterization and expression analysis of HAK K^+^ transport family in Ipomoea. 3 Biotech. 11, 3. doi: 10.1007/s13205-020-02552-3 33269187 PMC7695775

[B22] Kacovská K. Papoušková K. Masrati G. Rosas-Santiago P. Przeczková T. Žárská V. . (2026). Trafficking of the human Na(+)/H(+) antiporter NHA2 to the plasma membrane requires cornichon COPII cargo receptors. Protein Sci. 35, e70492. doi: 10.1002/pro.70492 41676957 PMC12895380

[B23] Kim M. J. Ruzicka D. Shin R. Schachtman D. P. (2012). The Arabidopsis AP2/ERF transcription factor RAP2.11 modulates plant response to low-potassium conditions. Mol. Plant 5, 1042–1057. doi: 10.1093/mp/sss003 22406475

[B25] Li W. Li M. Li S. Zhang Y. Li X. Xu G. . (2022). Function of rice high-affinity potassium transporters in pollen development and fertility. Plant Cell Physiol. 63, 967–980. doi: 10.1093/pcp/pcac061 35536598

[B24] Li M. Li W. Zeng Y. Lu X. Li X. Qu H. . (2025). Two AP2/ERF transcription factors coregulate OsHAK1 to modulate potassium and cesium uptake in rice. Plant Cell 37. doi: 10.1093/plcell/koaf205 40825066

[B26] Liu T. Zhang Y. Xie Y. Yang R. Yuan M. Li Y. . (2025). Impact of the potassium transporter TaHAK18 on wheat growth and potassium uptake under stressful K^+^ conditions. J. Plant Physiol. 307, 154459. doi: 10.1016/j.jplph.2025.154459 40020273

[B27] Luo M. Chu J. Wang Y. Chang J. Zhou Y. Jiang X. (2024). Positive regulatory roles of Manihot esculenta HAK5 under K^+^deficiency or high salt stress. Plants (Basel) 13. doi: 10.3390/plants13060849 38592853 PMC10974855

[B28] Maierhofer T. Scherzer S. Carpaneto A. Müller T. D. Pardo J. M. Hänelt I. . (2024). Arabidopsis HAK5 under low K^+^ availability operates as PMF powered high-affinity K^+^ transporter. Nat. Commun. 15, 8558. doi: 10.1038/s41467-024-52963-6 39362862 PMC11450230

[B29] Morita S. Tamba N. Shibasaka M. Sasano S. Kadoike T. Urase Y. . (2023). In planta evidence that the HAK transporter OsHAK2 is involved in Na^+^ transport in rice. Biosci. Biotechnol. Biochem. 87, 482–490. doi: 10.1093/bbb/zbad020 36804662

[B30] Nayyeripasand L. Garoosi G. A. Ahmadikhah A. (2021). Genome-wide association study (GWAS) to identify salt-tolerance QTLs carrying novel candidate genes in rice during early vegetative stage. Rice (N Y) 14, 9. doi: 10.1186/s12284-020-00433-0 33420909 PMC7797017

[B31] Nieves-Cordones M. Alemán F. Martínez V. Rubio F. (2010). The Arabidopsis thaliana HAK5 K+ transporter is required for plant growth and K+ acquisition from low K+ solutions under saline conditions. Mol. Plant 3, 326–333. doi: 10.1093/mp/ssp102 20028724

[B32] Peng L. Xiao H. Li R. Zeng Y. Gu M. Moran N. . (2023). Potassium transporter OsHAK18 mediates potassium and sodium circulation and sugar translocation in rice. Plant Physiol. 193, 2003–2020. doi: 10.1093/plphys/kiad435 37527483

[B33] Rajappa S. Krishnamurthy P. Kumar P. P. (2020). Regulation of AtKUP2 expression by bHLH and WRKY transcription factors helps to confer increased salt tolerance to Arabidopsis thaliana plants. Front. Plant Sci. 11, 1311. doi: 10.3389/fpls.2020.01311 32983201 PMC7477289

[B34] Rosas-Santiago P. Lagunas-Gómez D. Barkla B. J. Vera-Estrella R. Lalonde S. Jones A. . (2015). Identification of rice cornichon as a possible cargo receptor for the Golgi-localized sodium transporter OsHKT1;3. J. Exp. Bot. 66, 2733–2748. doi: 10.1093/jxb/erv069 25750424 PMC4986874

[B36] Shen L. Fan W. Li N. Wu Q. Chen D. Luan J. . (2023). Rice potassium transporter OsHAK18 mediates phloem K(+) loading and redistribution. Plant J. 116, 201–216. doi: 10.1111/tpj.16371 37381632

[B39] Shen Y. Shen L. Shen Z. Jing W. Ge H. Zhao J. . (2015). The potassium transporter OsHAK21 functions in the maintenance of ion homeostasis and tolerance to salt stress in rice. Plant Cell Environ. 38, 2766–2779. doi: 10.1111/pce.12586 26046379

[B38] Shen T. Xu F. Chen D. Yan R. Wang Q. Li K. . (2024). A B-box transcription factor OsBBX17 regulates saline-alkaline tolerance through the MAPK cascade pathway in rice. New Phytol. 241, 2158–2175. doi: 10.1111/nph.19480 38098211

[B35] Shen C. Yuan J. (2021). Genome-wide investigation and expression analysis of K^+^-transport-related gene families in Chinese cabbage (Brassica rapa ssp. pekinensis). Biochem. Genet. 59, 256–282. doi: 10.1007/s10528-020-10004-z 32990910

[B40] Siddique M. H. Babar N. I. Zameer R. Muzammil S. Nahid N. Ijaz U. . (2021). Genome-wide identification, genomic organization, and characterization of potassium transport-related genes in Cajanus cajan and their role in abiotic stress. Plants (Basel) 10. doi: 10.3390/plants10112238 34834601 PMC8619154

[B41] Singh A. Kanwar P. Yadav A. K. Mishra M. Jha S. K. Baranwal V. . (2014). Genome-wide expressional and functional analysis of calcium transport elements during abiotic stress and development in rice. FEBS J. 281, 894–915. doi: 10.1111/febs.12656 24286292

[B42] Song T. Shi Y. Shen L. Cao C. Shen Y. Jing W. . (2021). An endoplasmic reticulum-localized cytochrome b(5) regulates high-affinity K(+) transport in response to salt stress in rice. Proc. Natl. Acad. Sci. U.S.A. 118. doi: 10.1073/pnas.2114347118 34876526 PMC8685926

[B44] Šustr M. Konrádová H. Martinčová M. Soukup A. Tylová E. (2024). Potassium transporter KUP9 regulates plant response to K(+) deficiency and affects carbohydrate allocation in A.thaliana. J. Plant Physiol. 292, 154147. doi: 10.1016/j.jplph.2023.154147 38096629

[B45] Templalexis D. Tsitsekian D. Liu C. Daras G. Šimura J. Moschou P. . (2022). Potassium transporter TRH1/KUP4 contributes to distinct auxin-mediated root system architecture responses. Plant Physiol. 188, 1043–1060. doi: 10.1093/plphys/kiab472 34633458 PMC8825323

[B46] Uchiyama M. Fudaki R. Kobayashi T. Adachi Y. Ukai Y. Yoshihara T. . (2022). Rice OsHAK5 is a major potassium transporter that functions in potassium uptake with high specificity but contributes less to cesium uptake. Biosci. Biotechnol. Biochem. 86, 1599–1604. doi: 10.1093/bbb/zbac152 36085524

[B48] Wang Y. Wu W. H. (2013). Potassium transport and signaling in higher plants. Annu. Rev. Plant Biol. 64, 451–476. doi: 10.1146/annurev-arplant-050312-120153 23330792

[B49] Wang Y. Wu W. H. (2017). Regulation of potassium transport and signaling in plants. Curr. Opin. Plant Biol. 39, 123–128. doi: 10.1016/j.pbi.2017.06.006 28710919

[B50] Wang Y. Zhang Y. Wei Y. Meng J. Zhong C. Fan C. (2022). Characterization of HAK protein family in Casuarina equisetifolia and the positive regulatory role of CeqHAK6 and CeqHAK11 genes in response to salt tolerance. Front. Plant Sci. 13, 1084337. doi: 10.3389/fpls.2022.1084337 36816483 PMC9936244

[B47] Wang M. Zheng Q. Shen Q. Guo S. (2013). The critical role of potassium in plant stress response. Int. J. Mol. Sci. 14, 7370–7390. doi: 10.3390/ijms14047370 23549270 PMC3645691

[B51] Yang T. Zhang S. Hu Y. Wu F. Hu Q. Chen G. . (2014). The role of a potassium transporter OsHAK5 in potassium acquisition and transport from roots to shoots in rice at low potassium supply levels. Plant Physiol. 166, 945–959. doi: 10.1104/pp.114.246520 25157029 PMC4213120

[B52] Zeng P. Xie T. Shen J. Liang T. Yin L. Liu K. . (2024). Potassium transporter OsHAK9 regulates seed germination under salt stress by preventing gibberellin degradation through mediating OsGA2ox7 in rice. J. Integr. Plant Biol. 66, 731–748. doi: 10.1111/jipb.13642 38482956

[B55] Zhang M. L. Huang P. P. Ji Y. Wang S. Wang S. S. Li Z. . (2020b). KUP9 maintains root meristem activity by regulating K^+^ and auxin homeostasis in response to low K. EMBO Rep. 21, e50164. doi: 10.15252/embr.202050164 32250038 PMC7271654

[B56] Zhang M. Liang X. Wang L. Cao Y. Song W. Shi J. . (2019). A HAK family Na(+) transporter confers natural variation of salt tolerance in maize. Nat. Plants 5, 1297–1308. doi: 10.1038/s41477-019-0565-y 31819228

[B54] Zhang L. Sun X. Li Y. Luo X. Song S. Chen Y. . (2021). Rice Na^+^-permeable transporter OsHAK12 mediates shoots Na^+^ exclusion in response to salt stress. Front. Plant Sci. 12, 771746. doi: 10.3389/fpls.2021.771746 34950167 PMC8688356

[B53] Zhang H. Xiao W. Yu W. Jiang Y. Li R. (2020a). Halophytic Hordeum brevisubulatum HbHAK1 facilitates potassium retention and contributes to salt tolerance. Int. J. Mol. Sci. 21. doi: 10.3390/ijms21155292 32722526 PMC7432250

[B57] Zhang M. Zhou X. Wang L. Liang X. Liu X. Jiang C. (2025). A SnRK2-HAK regulatory module confers natural variation of salt tolerance in maize. Nat. Commun. 16, 4026. doi: 10.1038/s41467-025-59332-x 40301371 PMC12041354

[B58] Zhao S. Zhang M. L. Ma T. L. Wang Y. (2016). Phosphorylation of ARF2 relieves its repression of transcription of the K+ transporter gene HAK5 in response to low potassium stress. Plant Cell 28, 3005–3019. doi: 10.1105/tpc.16.00684 27895227 PMC5240742

[B59] Zhu H. Guo J. Ma T. Liu S. Zhou Y. Yang X. . (2023). The Sweet Potato K^+^ transporter IbHAK11 regulates K^+^ deficiency and high salinity stress tolerance by maintaining positive ion homeostasis. Plants (Basel) 12. doi: 10.3390/plants12132422 37446983 PMC10346689

